# Immunotherapy for Head and Neck Squamous Cell Carcinoma

**DOI:** 10.1007/s11912-018-0654-5

**Published:** 2018-03-03

**Authors:** Jessica Moskovitz, Jennifer Moy, Robert L. Ferris

**Affiliations:** 1 Department of Otolaryngology-Head and Neck Surgery, University of Pittsburgh, Pittsburgh, PA USA; 2 Department of Immunology, University of Pittsburgh, Pittsburgh, PA USA; 3 Cancer Immunology Program, University of Pittsburgh Cancer Institute, Pittsburgh, PA USA; 4 University of Pittsburgh Eye and Ear Institute, 200 North Lothrop Street, Suite 500, Pittsburgh, PA 15213 USA

**Keywords:** Checkpoint receptor, Immuno-oncology, PD1, CTLA4, Tumor infiltrating lymphocyte, Immune checkpoint receptor blockade

## Abstract

**Purpose of Review:**

Discussion of current strategies targeting the immune system related to solid tumors with emphasis on head and neck squamous cell carcinoma (HNSCC).This review will outline the current challenges with immunotherapy and future goals for treatment using these agents.

**Recent Findings:**

Agents targeting immune checkpoint receptors (IR) such as program death 1 (PD1) have been used in the clinical realm for melanoma and non-small cell lung cancer (NSCLC), and the use of these agents for these malignancies has provided crucial information about how and why patients respond or not to inhibitory checkpoint receptor blockade therapy (ICR). The anti PD1 agent, nivolumab, was recently approved by the FDA as a standard of care regimen for patients with platinum refractory recurrent/metastatic (R/M) HNSCC. Molecular pathways leading to resistance are starting to be identified, and work is underway to understand the most optimal treatment regimen with incorporation of immunotherapy.

**Summary:**

ICR has renewed interest in the immunology of cancer, but resistance is not uncommon, and thus understanding of these mechanisms will allow the clinician to appropriately select patients that will benefit from this therapy.

## Introduction

Head and neck squamous cell carcinoma (HNSCC) is the sixth most common cancer globally with a high mortality rate of 40 to 50% [1]. There is a high need for improved therapy in the locally advanced as well as the recurrent and metastatic (R/M) population. Until the introduction of immunotherapy agents, the only new agent that had been FDA approved for HNSCC in the USA was cetuximab, a monoclonal antibody (mAb) targeting epidermal growth factor receptor (EGFR). Despite initial excitement about this targeted agent, the addition of cetuximab to platinum -based chemotherapy resulted in only a 2.7-month survival increase with only a 20% reduction in the relative risk of death [2••]. This lackluster response is likely because of multiple systemic alterations that are required for carcinogenesis. For patients with R/M HNSCC with progressive disease after platinum-based therapy, prognosis is even worse with less than 5% surviving for a year [3].

Immunotherapy garnered enthusiasm because these agents use the patient’s own immune system, which can become suppressed by cancer cells, to fight the tumor. The hope was that by releasing suppressed immune cells and allowing these cells to be activated, immune cells could fight off tumor such as how the cells respond to an infection. Although immunotherapy has provided an option for cancer treatment to patients that previously had no options, clinicians and scientists are learning that the complex immune system is sculpted by cancer and that different types of cancers induce some changes that are similar but some changes that are different.

Although only a small percentage of patients respond to immunotherapy as monotherapy, often the response that are seen are durable and deep. The exciting results from treatment with these agents has led to an explosion in interest in the immune system and how to harness it to fight cancer. These new therapies are not without side effects, however, and some of the side effects have occurred many years after therapy cessation.

## Immunology of Cancer

### The Immune System

The immune system is divided into two parts: adaptive and innate. The adaptive immune system is comprised of T cells and B cells and involves a directed response resulting from recognition of specific antigens loaded on a major histocompatibility complex (MHC) molecule. T cell activation is the result of two signals within the context of a confirmatory third signal. Signal 1 occurs at the “immune synapse” where tumor antigens bound to the MHC molecule on the surface of antigen presenting cells (APCs) are presented to a T cell receptor (TCR). Signal 2 consists of either a co-stimulatory signal, such as the cluster of differentiation (CD) 28: B7 interaction, or an inhibitory signal [4]. The final signal, from immune activating cytokines such as interleukin 12 (IL12) or type I interferon (IFN), modulates the immune response, directing the cell towards inhibition or stimulation. Effective antigen presentation leading to T cell activation is enhanced and sustained by the induction of co-stimulatory cell surface molecules. To avoid an over-reactive immune system that leads to auto-immunity, immune cells express co-inhibitory receptors (immune checkpoints) that determine if a T cell is activated or becomes anergic, or nonresponsive, to the antigen displayed on the MHC molecule [5].

### The Immune System and Cancer

**Fig. 1:**
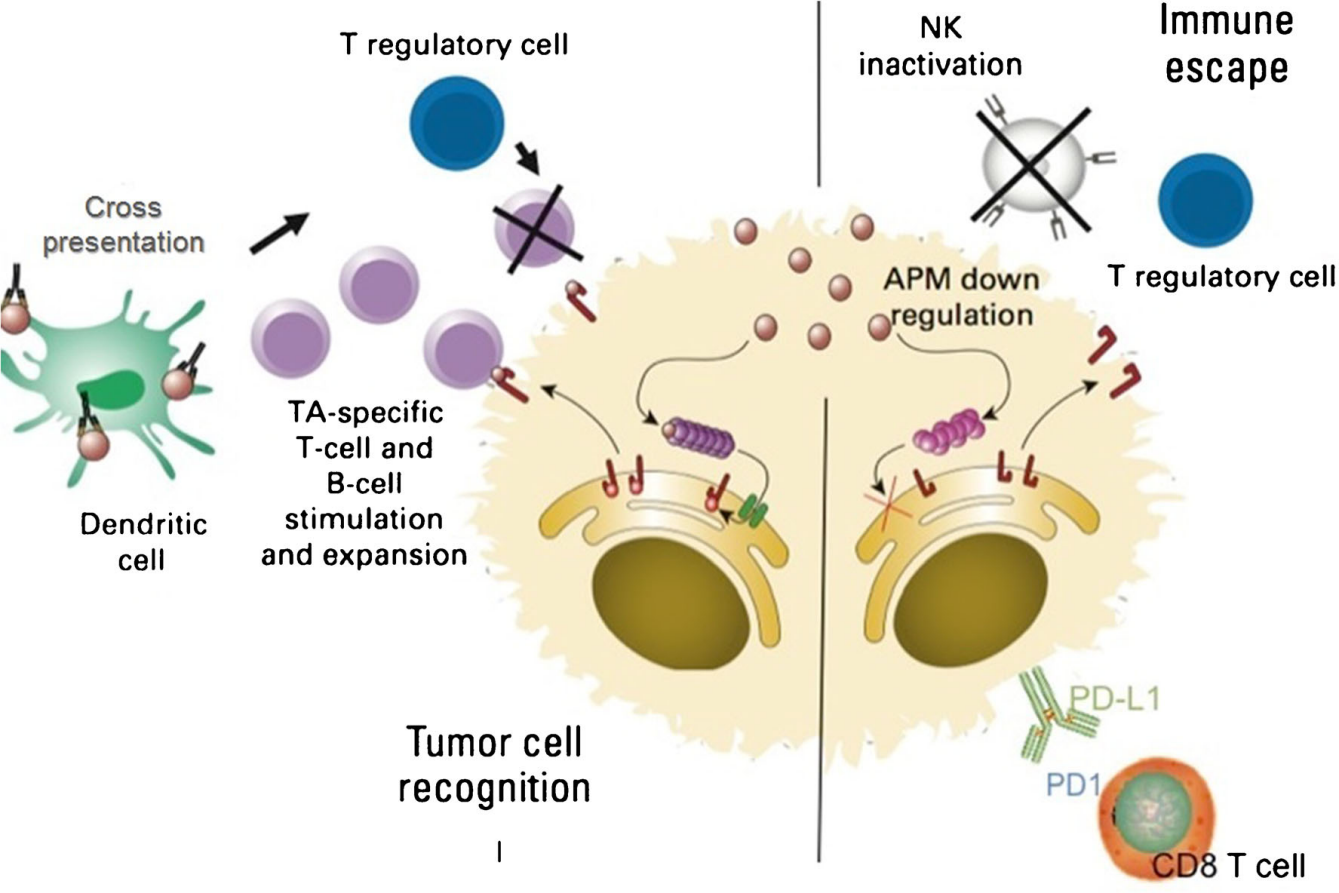
Mechanisms of immune escape. Processed tumor antigen is presented to T cells by antigen presenting cells such as dendritic cells. T cells and natural killer cells (NK) targeting tumor cells are thwarted by tumor “immunoediting” via downregulation of antigen processing machinery (APM). Intrinsic suppressive signals on tumor antigen specific T cells (checkpoint receptors such as PD1) as well as extrinsic suppressive signals from regulatory T cells (Treg) contribute to tumor immune escape

Ideally, the immune system recognizes tumor cells in a premalignant state and destroys these cells. However, tumor cells develop mechanisms to thwart immune recognition and response, a dynamic process termed immunoediting that leads to immune escape (Fig. 1) [6, 7]. Inhibitory checkpoint receptors (IR) expressed on activated immune cells such as cytotoxic T lymphocyte antigen 4 (CTLA4) and its ligands CD80 and CD86, and program death 1 (PD1) and its ligands PD-L1 and PD-L2, play an important role in the tumor microenvironment (TME) [8].Often expression of these inhibitory receptors signifies an exhausted T cell that has lost its normal function, including reduced proliferative capacity or cytolytic activity. However, this dysfunctional state can be reversed with IR blockade [9]. Resistance mechanisms to this T cell reinvigoration process may arise from expression of multiple IR or other acquired cellular changes [9, 10].

T cells recognize tumor antigen (TA) and produce interferon gamma (IFN-γ) that upon binding to its receptor, leads to signal transduction through the janus kinase (JAK1 and JAK2) and the signal transducer and activator of transcription (STAT) pathway. Interferon-stimulated gene expression resulting from this interaction has beneficial anti-tumor effects including improved antigen presentation and chemokine production [10, 11]. However, IFN-γ signaling also thwarts this anti-tumor effect through influencing inactivation of T cells by induction of PD-L1 expression [10, 12]. Mouse models have shown that events leading to lack of sensitivity to IFN-γ leads to acquired resistance to immunotherapy [9, 13]. This lack of sensitivity to IFN-γ results from loss of heterozygosity mutations in JAK 1 and 2, and similar to mouse models, these mutations were noted in melanoma patients that were resistant to anti-CTLA4 treatment [14]. Oncogenic signals, via the human epidermal growth factor receptor (HER) family members, may also modulate immune escape JAK-STAT signaling [15]. Prolonged IFN-γ stimulation has also been shown in mouse models suggest that STAT1 signaling leads to resistant tumors in a PDL1 independent manner [9].

Tumor infiltrating lymphocytes (TIL) are present in many solid tumor types, and infiltrate characteristics have been established as predictors of prognosis and disease-specific survival [5]. For example, in colorectal carcinoma, a higher CD8 TIL population and a lower CD4/CD8 ratio correlated with a prolonged disease-free survival [16]. Examination of the TME in multiple solid tumor types supports evidence that a T cell infiltrated tumor may have more prognostic significance than traditional staging [17, 18]. However, not all tumors are infiltrated with lymphocytes, and therefore these tumors may require additional agents to mobilize the lymphocyte population into the tumor tissue.

## Immunology of Head and Neck Squamous Cell Carcinoma

The immune system modifications noted in HNSCC patients suggest that this cancer is an overall immunosuppressive process. In the peripheral bloodstream, HNSCC patients have less overall number of white blood cells, which are comprised of a greater proportion of suppressive regulatory T cells (Treg). Additionally, TIL within HNSCC tumors are comprised of an even more suppressive population of Treg cells than in the peripheral bloodstream of HNSCC patients [19–22].

Human papillomavirus positive (HPV+) HNSCC tumors have one of the higher levels of infiltrating Tregs. Studies exploring the relationship of Treg infiltration to patient prognosis are varied; some show improved prognosis with a higher number of TIL Treg, [1], and others showing this benefit only with a high CD8/Treg ratio as seen with HPV+ disease [23]. High CD8+ TIL seen in HPV + disease has been shown in several studies to confer improved disease-free survival [5, 23, 24]. These cell populations express IR that can be targeted by inhibitory checkpoint receptor blockade therapy (ICR).

The results of several clinical trials have led to ICR agents such as pembrolizumab and nivolumab, PD1 blocking ICR agents, to be approved as standard of care for several solid tumors including HNSCC [25••, 26]. Patients refractory to standard platinum regimens and other cytotoxic therapies that previously had no other treatment options showed a response to nivolumab. However, the percentage of patients that respond to inhibitory checkpoint receptor blockade (ICR) is still unsatisfactory. The single arm, phase Ib Keynote 012 trial evaluated pembrolizumab, an anti-PD1 agent, in patients with R/M HNSCC. The primary endpoint of the study, overall response rate (ORR), was 18%. Only 9% of patients had grades 3 or 4 adverse events (AEs) [26]. This led to accelerated but limited approval of pembrolizumab for R/M HNSCC with a follow-up phase III study, Keynote 048. The response rate of the Keynote 012 trial was similar to the recently completed phase III Checkmate 141 randomized trial evaluating overall survival (OS) in 361 patients with platinum refractory R/M HNSCC treated with nivolumab or investigator’s choice (methotrexate, docetaxel, or cetuximab). The median OS was 7.5 months in the nivolumab group compared to 5.1 months for the investigator’s choice group [25••]. Grades 3 and 4 AEs were one third the rate of the control arm, thus contributing to improved quality of life for this patient population [25••].

## The Future of Immuno-Oncology

The future of immunotherapy holds exciting promise. Trials are underway that evaluate immunotherapy combined with current cytotoxic agents at different dose regimens as well as radiation therapy. Additionally, using multiple immune targeted agents concurrently has shown promise as a treatment strategy. Defining the appropriate regimen with the least toxicity and with durable responses are the goals of immunotherapy clinical trials today. Targeting redundant pathway mechanisms that lead to cancer progression will likely provide the best chance for curative therapy. A major therapeutic barrier remains for patients with poorly lymphocyte infiltrated tumors.

### Combination Immunotherapy Agents

Despite the enthusiasm regarding ICR, the majority of patients do not benefit from anti-PD1 therapy. Interest has turned to combining ICR agents with the hope of overcoming multiple layers of resistance to enhance efficacy in a synergistic manner, while maintaining an acceptable toxicity profile. It remains unclear how blockade of one immune checkpoint receptor affects other checkpoint receptors, and if blockade leads to cross talk downstream with other pathways. The use of combination immunotherapies has shown profound synergy and may lead to further treatment advances compared to use as a monotherapy or with current cytotoxic regimens.

Emerging trials are beginning to evaluate targeting checkpoint receptors other than PD1. Preclinical studies have identified several promising potential therapeutic targets, and many of these agents are being tested in combination trials with anti-PD1 therapy. CTLA4 and PD1 are considered to be non-redundant pathways, and this combination has been tested in melanoma patients confirming the synergism of blockade of these two IRs [27•]. Trials are underway evaluating this combination in other solid tumors. Other combinations are being tested in HNSCC (Table 1). There are several novel immuno-oncology agents in development; however, this is beyond the scope of this brief review article.

**Table 1:** Selected anti-PD1 + anti-CTLA4 combination immunotherapy trials

Clinical trial number (NCT)/acronym	Therapeutic agent	Phase	Patient eligibility	Status
NCT02551159/Kestrel	Durvalumab (MEDI4736) ± tremelimumab vs standard of care (SOC) EXTREME regimen (cetuximab + cisplatin/carboplatin + fluoruracil)	III	R/M HNSCC	Recruiting
NCT02369874/Eagle	Durvalumab (MEDI4736) ± tremelimumab vs standard of care	III	R/M HNSCC (PDL1 ± )	
NCT02741570/CheckMate-651	Nivolumab + ipilimumab vs SOC EXTREME regimen	III	R/M HNSCC	Recruiting
NCT02823574/CheckMate-714	Nivolumab + ipilimumab vs Nivolumab + ipilimumab placebo	II	R/M HNSCC	Recruiting

### Immunotherapy and Radiation

Historically, radiation therapy (RT) has been considered an immunosuppressive treatment modality with the mechanism of cell death related to direct DNA damage [28, 29]. In vitro studies of (RT) triggering immunogenic cell death (ICD), a process that converts the irradiated tumor into an in situ vaccine [30]. One of the proposed theories regarding the potential advantage to radiation with immunotherapy is that ICD can potentially enhance systemic responses through an “abscopal effect” where local therapy induces a systemic response that lasts beyond the completion of RT treatment [31]. These changes could alter the TME making it more responsive to PD1 pathway blocking agents. Preclinical abscopal responses have demonstrated the additive effects of RT with anti-PD1 therapy [28].Similar to PD1, preclinical studies have also noted synergism of RT with anti-CTLA4 agents [28, 32•]. Ionizing radiation stimulates the adaptive immune response through several other mechanisms, any of which may be synergistic with immunotherapy Additionally, RT has been shown to induce upregulation of PD-L1 on both tumor cells and MDSC [33]. Preclinical models have reported various techniques and dosing schedules for different tumor models, and thus it is of paramount importance to determine the radiation dose and fractionation for inducing an optimum immune response [28, 34]. Trials are ongoing exploring the synergistic potential of RT and immunotherapy (Table 2).

**Table 2:** Selected combination radiation with immunotherapy trials

	Clinical trial number (NCT)/acronym	Therapeutic agent	Phase	Patient eligibility	Status
Anti-PD1					
	NCT02952586/JAVELIN 100	Avelumab + cisplatin/RT vs cisplatin/RT alone	III	Locally advanced HNSCC	Recruiting
	NCT03040999/KEYNOTE-412	Pembrolizumab + chemo/RT vs chemo/RT alone	III	Locally advanced HNSCC	Recruiting
	NCT0276459/RTOG 3504	Cisplatin/RT ± nivolumab	III (with phase 1 lead in)	Intermediate to high risk HNSCC	Recruiting
	NCT02641093	Adjuvant cisplatin/pembrolizumab/RT	II	Surgically resected, high risk (+ margin and/or ECS)	Recruiting
	NCT02777385	Concurrent vs sequential pembrolizumab combined with cisplatin/IMRT	II	Previously untreated, intermediate to high risk HNSCC	Recruiting
	NCT02892201	Pembrolizumab	II	Biopsy proven residual HNSCC within 24 weeks post RT (± systemic cytotoxic chemo)	Recruiting
	NCT03085719	Pembrolizumab with high vs high and low dose RT	II	R/M HNSCC with progressive or stable disease on prior anti-PD1 therapy	Not yet recruiting

### Immunotherapy and Cytotoxic Chemotherapy

Traditionally, systemic chemotherapy was thought to impart its effects solely through direct tumor killing. However, recent studies have shown significant immune stimulation with lower doses of systemic cytotoxic therapy [35].Chemotherapy triggers both the adaptive and innate immune system through several modalities including promoting cellular changes to dying cancer cells rendering them recognizable by the immune system. Cytotoxic chemotherapy affects bone marrow hematopoiesis and associated myeloid cell mobilization; however, at lower dosages of agents such as cisplatin, there is an increase in the antigen presenting population of dendritic cells (DC) and elimination of the suppressive MDSC [36]. Mouse models of esophageal squamous cell carcinoma (SCC) treated with cisplatin and 5- fluorouracil (5-FU) had increased percentages of intratumoral CD4 and CD8 [37]. Other preclinical models have shown an increase in T helper 1 (TH1) cytokines such as IFN-γ and interleukin (IL)-2 [38]. These agents may, in the future, become increasingly important as adjuncts to immunotherapy for patients with tumors that are poorly infiltrated with lymphocytes.

## Side Effects and Long-Term Disease Monitoring

### Side Effects

The toxicities associated with immunotherapy differ from traditional systemic therapy. The majority of these side effects are autoimmune as compared to renal failure and anemia which is seen with standard cytotoxic therapy. The most well-known side effects seen with the anti-CTLA4 agent ipilimumab include pneumonitis and colitis [39]. Nivolumab and other anti-PD1 agents have a more favorable side effect profile compared to ipilimumab. In the checkmate 141 trial, autoimmune endocrinopathies were the most common side effect, and often this did not require cessation of the immunotherapy agent. Additionally, for this trial, patients had one third the rate of grades 3 and 4 AEs with nivolumab compared to cytotoxic therapy [25••].

### Disease Monitoring

#### Prognostic Biomarkers

Identifying patients that would benefit from immunotherapy prior to starting treatment would eliminate subjecting patients to autoimmune side effects who will not benefit from ICR. Potential biomarkers of disease response, such as PD-L1, are actively being evaluated. Studies evaluating PD-L1 as a biomarker for disease response thus far, however, have yielded somewhat mixed results. This may be due to the non-standardized definition of a positive threshold with immunohistochemistry between different lab detection kits used. Positive results range anywhere from over 1 to over 50% of cells staining for PD-L1. Also, some studies have evaluated the concentration of PD-L1 on tumor cells and peritumoral tissue while others evaluated PDL1 on tumor cells only. However, PD-L1 expression is induced by IFN-γ as a mechanism for tissue protection. Therefore, evaluation of PD-L1 and IFN-γ may represent a way to determine the presence of infiltrating TIL as well as provide a method to predict patient response to immunotherapy [40].

#### Response Criteria and Imaging

Monitoring strategies used for responders to cytotoxic chemotherapy may not be applicable for immunotherapy. RECIST criteria used for cytotoxic agents is based on the premise that an agent that works on a tumor results in shrinkage of the tumor, and tumors resistant to an agent enlarge. In contrast to cytotoxic agents, immunotherapeutic agents have shown to produce responses with an assortment of kinetic patterns, even including transient tumor swelling [41]. This “tumor flare” response, represented by increased tumor diameter radiographically, may be due to lymphocytic infiltration of tumor. Additionally, this tumor flare may be a result of delayed immune cell activation during which time the tumor may grow while the immune system is preparing an anti-tumor response. The use of standard radiographic criteria may lead to erroneous cessation of the immunotherapeutic agent in this scenario. Because of this phenomenon, alternative disease response endpoints are necessary for immunotherapeutic agents. However, it should be mentioned that the “flare” is quite uncommon in HNSCC [42–44].

## Conclusions

Understanding the complex balance of immune cell interactions and cell signaling has advanced significantly and has led to renewed interest in immunotherapy as a potential cure for multiple solid tumor types, including HNSCC. It is of paramount importance however, that rational clinical trial designs are developed to identify and prevent potentially serious autoimmune reactions and other AE. Current results from immunotherapy trials have shown for the first time an improved response rate and overall survival in HNSCC patients with R/M disease.
